# Prognostic Significance of the Dynamic Change of Programmed Death-ligand 1 Expression in Patients with Multiple Myeloma

**DOI:** 10.7759/cureus.4401

**Published:** 2019-04-06

**Authors:** Jian Guan, Renching Wang, Syed Hasan, Luwei Tao, Mohammed Wazir, Akriti G Jain, Xiang Zhu, Sherrie Perkins, Salama Mohamed, Chung-Che Chang, Shahram Mori

**Affiliations:** 1 Internal Medicine, Florida Hospital, Orlando, USA; 2 Pathology, Taichung Veteran General Hospital, Taichung, TWN; 3 Miscellaneous, Florida Hospital, Orlando, USA; 4 Pathology, University of Utah, Salt Lake City, USA; 5 Pathology, Mayo Clinic, Rochester, USA; 6 Pathology, Florida Hospital, Orlando, USA; 7 Oncology, Florida Hospital, Orlando, USA

**Keywords:** dynamic change, prognosis, multiple myeloma, immunotherapy

## Abstract

Background

The inhibition of programmed cell death protein 1/programmed death-ligand 1 (PD-1/PD-L1) signaling pathway has been shown to be an effective targeted therapy in fighting both solid organ cancers and hematological malignancies. PD-L1 expression also serves as a prognostic marker in various cancers. However, the expression of PD-L1 and its prognostic significance in multiple myeloma remains largely unknown.

Methods

Immunohistochemistry staining of PD-L1 was performed in bone marrow biopsy samples (total of 85 samples) in 32 myeloma patients receiving autologous stem cell transplant (ASCT) at various time points: before ASCT, post-ASCT, and/or at relapse after ASCT. More than 1% of myeloma cells with PD-L1 staining was considered a positive expression of PD-L1. A correlation analysis was performed between post-ASCT overall survival (OS) and the status of PD-L1 expression.

Results

In this pilot study, a total of 11 patients (34%) out of our cohort (32 patients) were positive for PD-L1 expression at least once during the course of the disease. A dynamic change of PD-L1 expression was noted in three patients converting from negative (before ASCT) to positive (post-ASCT) and two patients converting from positive (before ASCT) to negative (post-ASCT). Patients with positive PD-L1 expression persisting or occurring post-ASCT had shorter post-ASCT overall survival than those with negative PD-L1 expression post-ASCT (median survival: 13 vs 23 months, p<0.05). No significant differences were detected in the known prognostic factors between these two groups at the time of ASCT. Pre-transplant PD-L1 expression status, however, showed no significant impact on post-ASCT overall survival. Furthermore, a few patients switching from positive PD-L1 expression before ASCT to negative PD-L1 expression post-ASCT had a relatively good post-ASCT overall survival (n=2, overall survival of 29 and 56 months, respectively).

Conclusion

Immunohistochemistry can be reliably used for measuring PD-L1 expression in decalcified marrow core biopsy materials. Our results suggest that positive PD-L1 expression persisting/occurring post-ASCT could be an adverse prognostic marker for post-ASCT OS. Additionally, PD-L1 expression appears to be dynamic and is subjected to change after ASCT. Our findings suggest that periodically monitoring PD-L1 expression in patients with multiple myeloma post-ASCT is warranted. Further studies are needed to confirm our initial observation and to evaluate if timely intervention with PD-L1 blockade can improve post-ASCT outcomes in myeloma patients.

## Introduction

Multiple myeloma is a B cell malignancy producing monoclonal immunoglobulin and leading to organ damage, including kidney injury, lytic bone disease, hypercalcemia, and anemia [1]. Accounting for 1.8% of all malignancies, multiple myeloma is the second most common hematological neoplasm in developed countries.Multiple myeloma mainly affects elderly populations with the average age at diagnosis approximating 70 years. Multiple myeloma used to be considered a dismal and incurable disease associated with an extremely poor prognosis. However, the median survival of patients with multiple myeloma was dramatically improved after the introduction of high dose chemotherapy with autologous stem cell transplant (ASCT) [2]. In the past two decades, the emergence of novel agents, such as proteasome inhibitors, immunomodulatory drugs, and monoclonal antibodies, against antigens expressed on multiple myeloma cells have further improved the management of this disease [3]. Despite these advances in treatment options, it is still challenging to manage this disease, as most multiple myeloma in most patients continues to relapse or progress after initial remission, resulting in the development of relapsed/refractory multiple myeloma (RRMM) [4]. RRMM faces even poorer outcomes, which is further complicated by treatment-related toxicities [4].

The membrane-bound molecules programmed cell death protein 1 (PD-1) and its ligand programmed death-ligand 1 or 2 (PD-L1 or PD-L2) belong to the immune checkpoint pathway [5]. The binding of PD-1 to PD-L1 or PD-L2 downregulates antigen-stimulated T cell proliferation and cytokine production and inhibits cytotoxic T-lymphocyte mediated killing [5]. This results in T-cell anergy and apoptosis, leading ultimately to lymphocyte exhaustion and immune evasion. The upregulation of the PD-1/PD-L1 pathway by cancer cells can contribute to immune escape and tumor proliferation, whereas inhibitors of these checkpoints can induce immune activation and immune-mediated tumor regression [6].

In recent years, researchers have been studying and evaluating the PD-1/PDL-L1 blockade as a potential target for the treatment of multiple myeloma [7-8]. Studies reported that PD-L1 is absent from normal plasma cells but is expressed on pathological plasma cells from myeloma patients [7]. However, the expression of PD-L1 and its prognostic significance in multiple myeloma remain largely unknown. The goal of this pilot study was to explore the dynamic expression and prognostic values of PD-L1 expression via immunohistochemical studies of bone marrow samples from patients with multiple myeloma receiving autologous stem cell transplant from a single institution.

## Materials and methods

Patients

This retrospective study was conducted on a total of 32 patients with multiple myeloma who received ASCT at the Florida Hospital Blood & Marrow Transplant Center from 2008 to 2013 and were deceased before December 2017. We were only able to obtain Institutional Review Board (IRB) of Florida Hospital approval for using samples of deceased patients without additional consent. All patients were diagnosed according to the International Myeloma Working Group (IMWG) criteria. All patients included in this study received similar chemotherapy regimens and underwent at least one ASCT, had a minimum of a three-month follow-up post-ASCT available for performing PD-L1 immunohistochemical staining.

The clinical data and outcomes were obtained from the patients’ medical records and Florida Hospital Cancer Registry, including the patients’ age, gender, free light chain concentration/ratio, hemoglobin, platelet count, calcium level, albumin, b2-microglobulin, and survival time from stem cell transplant. These data were categorized according to the status of PD-L1 expression post-ASCT (Table 1).

**Table 1:** Clinical characteristics of patients at the time of the ASCT evaluation (all values are mean ± SE, unless otherwise indicated) Hemoglobin (Hgb), Lactate dehydrogenase (LDH), light chain (LC), Autologous stem cell transplant (ASCT), Standard error (SE)

Characteristics	PD-L1 Negative Post-ASCT (n=23)	PD-L1 Positive Post-ASCT (n=9)	p-value
Age (year)	57.2 ± 1.6	55.7 ± 3	0.639
Gender, female (%)	7 (30.4)	6 (66.7)	0.061
Hgb (g/dL)	10.8 ± 0.5	9.9 ± 0.6	0.318
Platelet (10^6/dL)	208.3 ± 20	170.6 ± 36.5	0.344
Albumin (g/dL)	3.4 ± 0.2	3.4 ± 0.2	1.000
Calcium (mg/dL)	9.5 ± 0.4	8.8 ± 0.1	0.289
b2-microglobulin (mg/L)	3.5 ± 0.6	2.8 ± 0.8	0.524
LDH (units/L)	174.8 ± 12.9	224.8 ± 37.9	0.118
LC type, Kappa (%)	15 (65.2)	7 (77.8)	0.491
LC ratio (Kappa/Lambda)	235.4 ± 194.9	125 ± 84	0.732

Immunohistochemistry staining

A total of 85 bone marrow biopsy specimens from these 32 patients at various time points (at the time of diagnosis, before ASCT, post-ASCT, and/or at relapse post-ASCT) were evaluated for this study. The hematoxylin and eosin (HE)-stained slides and syndecan-1 (CD138)-stained slides, which highlighted the plasma cells, of all samples were reviewed to confirm the diagnosis and the adequacy for the immunohistochemistry (IHC) staining of PD-L1. The PD-L1 stain was performed using Food and Drug Administration (FDA) approved monoclonal antibody (clone: SP142, Ventana Medical Systems, Inc, Tucson, Arizona) according to the manufacturer’s protocol, using decalcified formalin-fixed paraffin-embedded tissue sections. The PD-L1 stained slides of each case were scored by two pathologists independently without knowing any clinical data. The final score was the average score of the two pathologists. Greater than 1% of plasma cells with PD-L1 staining was considered positive for PD-L1 expression. The PD-L1 staining was mainly located in the cytoplasm and/or membrane. In addition to myeloma cells, PD-L1 expression was also detected in some tumor-infiltrating lymphocytes.

Statistical analysis

A T-test was performed to compare the difference in continuous variables, and χ2 was used to compare the difference in count numbers between PD-L1 negative and PD-L1 positive patients. A Kaplan Meier estimator was used to assess the survival time, and a log-rank test was used to compare the overall survival (OS) between PD-L1 negative and PD-L1 positive patients. All analyses were performed by using Stata version 14 (StataCorp., 2015). All 𝑃 values were two-tailed, and 𝛼< 0.05 was set as the level of statistical significance for all tests.

## Results

Patients

In this cohort, a total of 32 patients with relapsed multiple myeloma who underwent chemotherapy and stem cell transplant from Florida Hospital were studied (13 females and 19 males). The median age of patients was 56 years (ranging from 44 to 70). The post-ASCT follow-up period ranged from five months to 88 months with a median of 23 months.

PD-L1 expression during the disease course

**Figure 1:**
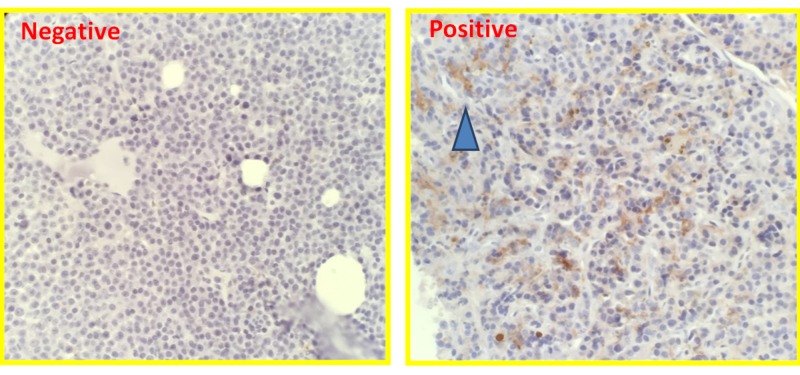
PD-L1 staining in multiple myeloma patients (PD-L1 expression was shown with dark brown staining in the right panel, as indicated by the blue arrow) Programmed death-ligand 1 (PD-L1)

PD-L1 expression was positive in five out of 26 (19.2%) patients (excluding six patients without pre-transplant staining) prior to the ASCT and nine out of 32 (28.1%) patients post-ASCT with the cutoff of 1% of plasma cells expressing PD-L1. Examples of PD-L1 expression via immunohistochemical staining are shown in Figure 1. Overall, 11 patients out of this cohort (34.3%) showed positive PD-L1 expression at least once over the disease course. A dynamic change of PD-L1 expression was observed in five patients. Two patients with a positive expression of PD-L1 prior to ASCT became negative post-ASCT. Additionally, three patients with negative PD-L1 expression prior to ASCT became PD-L1 positive post-ASCT. See Table 2 for details of dynamic changes in this cohort.

**Table 2:** Dynamic change of PD-L1 expression in patients with multiple myeloma Programmed death-ligand 1 (PD-L1), Autologous stem cell transplant (ASCT) The overall post-ASCT survival for two patients with initial positive PD-L1 expression prior to the ASCT but becoming negative post-ASCT is 42 months and 29 months, respectively. The overall post-ASCT survival for three patients with initial negative PD-L1 expression prior to the ASCT but becoming positive post-ASCT is 12 months, 22 months, and 40 months, respectively.

	PD-L1 Negative Post-ASCT	PD-L1 Positive Post-ASCT
PD-L1 Negative Pre-ASCT	18	3
PD-L1 Positive Pre-ASCT	2	3
No data Pre-ASCT	3	3
Summary	23	9

Prognostic significance of PD-L1 expression prior and post-ASCT

**Figure 2:**
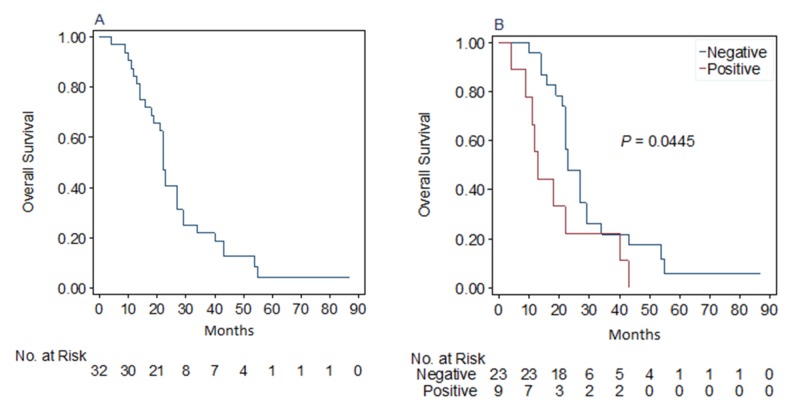
A: Overall survival of this cohort; B: Comparison of overall survival of patients with positive or negative PD-L1 expression post-ASCT Programmed death-ligand 1 (PD-L1), Autologous stem cell transplant (ASCT)

The median overall survival of this cohort post bone marrow transplant was 22 (18-27) months (Figure 2A). The much shorter overall survival of this cohort than reported in the literature is due to the limitation of the current study, which only used deceased patients’ marrow specimens, as approved by the IRB. The status of PD-L1 expression prior to ASCT did not impact the post-ASCT overall survival (data not shown).

Of note, the patients with positive PD-L1 expression post-ASCT showed shorter survival than those without PD-L1 expression (median post-ASCT overall survival: 13 months vs 23 months, p<0.05, Figure 2B). No significant differences in known prognostic parameters were observed between the PD-L1 positive and negative groups (Table 2). Additionally, the two patients with initial positive PD-L1 expression prior to ASCT but becoming negative post-ASCT showed overall survival (29 months and 56 months respectively) similar to that of patients with PD-L1 negative expression post-ASCT.

## Discussion

Our study, to the best of our knowledge, is the first description that PD-L1 expression in multiple myeloma patient bone marrow is a dynamic process and this dynamic change of PD-L1 expression may have prognostic significance. The changes in the bone marrow microenvironment following ASCT may play an essential role in the dynamic changes observed. Previous studies have shown that PD-L1 expression is under complicated regulation. Liu reported that interferon-gamma (INF-gamma) is a potent inducer and that multiple myeloma cells exhibited exaggerated upregulation of PD-L1 under INF-gamma induction as compared to normal plasma cells [8]. More recently, dynamic changes in PD-L1 expression have been reported in solid tumors after radiation [9-10].

Our findings suggest that patients with positive PD-L1 expression post-ASCT, but not prior to ASCT, have significantly shorter survival in our limited cohort. Additionally, we observed two patients who had a switch from PD-L1 positive expression prior to ASCT to PD-L1 negative expression. These two patients appear to have improved outcomes. Although it is difficult to assume any significance from two patients, nevertheless, taken together, these results suggest that PD-L1 expression in bone marrow has the potential to serve as a prognostic marker after ASCT. Monitoring PD-L1 expression in bone marrow for patients receiving ASCT could be employed to identify patients who are at high risk for poor outcomes, requiring additional treatment if these findings are confirmed by other, larger studies. Currently, no clinical trials have been published to demonstrate the efficacy of using PD-L1 inhibitors in treating patients with refractory multiple myeloma. Monitoring PD-L1 expression could be helpful to identify those high-risk patients during treatment.

Similar to our findings, previous studies [11-12] showed that the serum soluble PD-L1 level in multiple myeloma patients was a negative prognostic factor. This finding also suggested that the activation of the PD-L1 pathway correlates with disease relapse. Crescenzi et al. evaluated the expression of PD-L1 with immunohistochemistry staining in extra-medullary multiple myeloma lesions and suggested a possible link to poor prognosis [13]. However, the measurement of soluble PD-L1 is not available in the vast majority of clinical laboratories. In contrast, immunohistochemistry staining for PD-L1 is accessible for many clinical laboratories, making this method more applicable in routine clinical care.

Several mechanisms may attribute to the adverse outcomes of PD-L1 positive multiple myeloma patients post-ASCT. First, the PD-1/PD-L1 pathway negatively regulates T cell antigen receptor signaling. The expression of PD-L1 in multiple myeloma cells can serve as a mechanism for multiple myeloma cells to escape the host immune response [14-17]. Secondarily, studies have shown that expression of PD-L1 confers a proliferation advantage for MM cells by activating the phosphoinositide 3-kinases (PI3K) signaling pathway, which induces resistance to antimyeloma chemotherapy, including melphalan and bortezomib [15].

Our study has two limitations. First, this study is a retrospective analysis with a limited sample size. Second, the study is further limited to only studying deceased patients after ASCT (as per our IRB policies). These limitations have likely biased the study toward a cohort of patients with high-risk disease and poorer outcomes. However, even within this group of patients, PD-L1 expression appears to be an important prognostic marker post-ASCT. Further studies are needed to confirm our initial observations, study if this observation is limited to post-ASCT patients or is generalizable to all MM patients, and evaluate if a checkpoint blockade may have a role in improving patient outcomes in the post-ASCT setting, particularly for those patients with positive PD-L1 expression.

## Conclusions

Immunohistochemistry staining can be reliably used for measuring PD-L1 expression in bone marrow core biopsy materials. Positive PD-L1 expression persisting/acquired post-ASCT could be an adverse prognostic marker for post-ASCT OS. Additionally, PD-L1 expression appears to be dynamic and is subjected to change after ASCT. Our findings suggest that periodically monitoring PD-L1 expression in patients with multiple myeloma post-ASCT is warranted.
